# Pantothenic Acid, Vitamin C, and Biotin Play Important Roles in the Growth of *Lactobacillus helveticus*

**DOI:** 10.3389/fmicb.2018.01194

**Published:** 2018-06-04

**Authors:** Chunxiao Yao, Jiandong Chou, Tao Wang, Hongfei Zhao, Bolin Zhang

**Affiliations:** ^1^College of Biological Sciences and Technology, Beijing Forestry University, Beijing, China; ^2^Beijing Key Laboratory of Food Processing and Safety in Forestry, Beijing, China

**Keywords:** *Lactobacillus helveticus*, vitamins, fluorescent quantitative PCR, transcriptome, high density culture

## Abstract

*Lactobacillus helveticus* is an important lactic acid bacterium. The strains used in this study have proven probiotic function, and the potential to produce functional dairy products and bioactive peptides. To explore the effects of vitamins on the growth of *L. helveticus*, a chemically defined medium was designed and nine vitamins were tested. Pantothenic acid (Vb_5_), vitamin C (Vc), and biotin were necessary for the growth of *L. helveticus* CICC 22171. These three vitamins had an important effect on the glucose metabolism and energy metabolism of strain CICC 22171. Through transcriptomic analysis, we found that three vitamins were related to the synthesis of fatty acids and participate in the energy supply of the cells. Additionally, Vb_5_ was involved in the metabolism of bacterial proteins and lipids and was related to the activity of various enzymes. The results indicated that Vc was involved in protein metabolism, and biotin affected the intracellular transport mechanism of bacteria. The ability of vitamins to promote the growth of the strain was verified in skim milk medium. The results indicated that Vc, biotin, and Vb_5_ could promote the proliferation of *L. helveticus* but had no significant effect on *Lactobacillus bulgaricus*.

## Introduction

Lactic acid bacteria (LAB) are very important microorganisms in the food industry. The main feature of LAB is their ability to ferment saccharide substances to lactic acid (Jin, 1998). LAB are heterotrophic bacteria that need carbon, nitrogen, and growth factors for their growth. *Lactobacillus helveticus* (*L. helveticus*), which belongs to LAB group, was first isolated from cheese products. It is widely used in food industry, especially in dairy products such as Swiss cheeses (Emmental, Comte) and Italian cheeses (Parmigiano Reggiano, Grana Padano, and Provolone) (Vinderola et al., 2007). *L. helveticus* plays an important role in probiotic and nutraceutical food products as health-promoting culture. It also has the potential to produce bioactive peptides or bacteriocins in fermented dairy products when associated with prebiotics. It can therefore be considered as a multifunctional LAB in the food industry (Giraffa, 2014).

Because of the highly efficient proteolytic systems and bioactive peptide production capacity of *L. helveticus*, an increasing number of studies have focused on the proliferation of *L. helveticus* (Zhao et al., 2014). Skim milk culture medium with added lactose significantly promoted the growth of *L. helveticus*, and an effect of carbon source on the growth and metabolism of the strain was observed (Zhao, 2014). The addition of beet molasses and yeast extract also improved lactic acid production by *L. helveticus* CNRZ 303 (Chiarini et al., 1992). Liao (2009) added 18% of the tomato juice into the medium, and the viable count of *L. helveticus* reached 3.59 × 10^9^. Furthermore, the addition of wort to the culture medium also promoted the growth of *L. helveticus* (Wu et al., 2008). Not only that, there are some other studies that have focused on the high-density culture of *L. helveticus* (Liang, 2015; Liu, 2016). The factors that promote the proliferation of *L. helveticus* are mainly concentrated on carbon sources, nitrogen sources, and growth factors. Great number of studies have focused on carbon sources and nitrogen sources, and there has no more research on vitamins, amino acids, and other growth factors. The addition of tomato juice or other plant extracts in medium promotes the proliferation of strains, although these additives are mixtures and which chemical compound contributed to the proliferation of the strain is unknown. Vitamins are important growth factors that need to be added to the medium. It plays an important role in the growth of strains, but their role in microbial proliferation is unclear. The objective of this study was to: (i) determine vitamin requirements of *L. helveticus* CICC 22171; (ii) explore the mechanism of vitamins on the proliferation of strain CICC 22171; and (iii) study on whether the proliferation of vitamins can be extended to other strains.

## Materials and Methods

### Bacterial Strains and Growth Conditions

*Lactobacillus helveticus* CICC 22171 was separated from traditional Xinjiang cheese by the microbiology laboratory of Beijing Forestry University and preserved in the China Center of Industrial Culture Collection (CICC). *Lactobacillus delbrueckii* subsp. *bulgaricus* (*L. bulgaricus*) LB4 was provided by CICC. *L. helveticus* CGMCC 1.1877 was purchased from China General Microbiological Culture Collection Center (CGMCC). The strains were cultured in DeMan-Rogosa-Sharpe (MRS) medium for 18 h at 37°C.

### Vitamin Requirements of *L. helveticus* CICC 22171

In order to investigate the influence of vitamins on the proliferation of strain CICC 22171, a chemically defined medium (CDM) was designed. The details are shown in **Table 1**. The medium was sterilized at 121°C for 15 min, and then aneurin (Vb_1_), riboflavin (Vb_2_), pantothenic acid (Vb_5_), pyridoxal (Vb_6_), folic acid (Vb_9_), vitamin C (Vc), biotin, nicotinic acid, and 4-aminobenzoic acid were added to the CDM after sterile filtration (0.22-μm-pore-size filter). The control check (CK) group used complete CDM. The vitamin-deficient medium refers to a medium in which a specific vitamin was absent from the CDM. For example, CDM lacking Vc was named “Vc group.” The number of viable cells was measured by colony forming units in MRS medium.

**Table 1:** Composition of chemically defined medium.

Constituent	Concn (g/liter)	Constituent	Concn (g/liter)
Casein acid hydrolysate vitamin free	15	Aneurin (Vb_1_)	0.001
Glucose	20.0	Riboflavin (Vb_2_)	0.001
MgSO_4_	0.5	Vitamin b5 (Vb_5_)	0.001
MnSO_4_	0.25	Pyridoxal (Vb_6_)	0.002
K_2_HPO_4_	2.0	Folic acid (Vb_9_)	0.001
Tween-80	1.0	Vitamin C (Vc)	0.002
4-Aminobenzoic acid (PABA)	0.002	Biotin	0.001
Nicotinic acid	0.001		

### The Mechanism of Vitamins on the Proliferation of Strain CICC 22171

#### The Effects of Vitamins on Glucose Metabolism

*Lactobacillus helveticus* was cultured at 37°C for 18 h in MRS medium. Cells were harvested by centrifugation (4°C, 6000 *g*, 5 min), and then washed twice with an equal volume of sterile saline. After that, the precipitate was collected and suspended in an equal volume of sterile saline. Bacterial protein were extracted from activated cells using the One-step Bacterial Active Protein Extraction Kit (Sangon Biotech Shanghai Co., Ltd., Shanghai, China). Protein content was determined using the Coomassie Brilliant Blue method (Feng et al., 2010). Hexokinase (HK), phosphofructokinase (PFK), pyruvate kinase (PK), and lactate dehydrogenase (LDH) play important roles in metabolic pathways, and the activity of these enzymes was determined using a kit (Suzhou Keming Biotechnology Co., Ltd., Suzhou, China).

The gene expressions of HK, PFK, PK, and LDH were detected by the RT-qPCR method, and the experimental design refers to the MIQE guidelines (Bustin et al., 2009). Total RNA was extracted using the TRIzol kit (Sangon Biotech Shanghai Co., Ltd., Shanghai, China) and treated with RNase-free DNase. A total of 500 ng RNA samples were reverse-transcribed with an AMV First Strand cDNA Synthesis Kit (Sangon Biotech Shanghai Co., Ltd., Shanghai, China). cDNA products were used for SYBR Green-based RT-qPCR analysis, each sample was run in triplicate. The RT-qPCR conditions were as follows: 95°C for 3 min, followed by 40 cycles of 95°C for 7 s, 57°C for 10 s, and 72°C for 15 s, with a final step of 72°C for 10 min. Using CK group as a standard, the 16S rRNA gene was used as an internal reference, and the primer sequences were shown in **Table 2**. The expression levels of DEGs were calculated using the 2^-ΔΔ^*^C^*^t^ method (Livak and Schmittgen, 2001).

**Table 2:** Primer sequences.

16S-F: 5′-TGTAGCGGTGGAATGCGTAG-3′	16S-R: 5′-ATCTAATCCTGTTCGCTACCCA-3′
HK-F: 5′-TTCCTGCTCATCCCCTACTCA-3′	HK-R: 5′-CGATAACATTTTGAGCACCCAT-3′
PFK-F: 5′-GATTGGCTTTGATTCTGCTTG-3′	PFK-R: 5′-ACCACATTCACGTCCCATTAC-3′
PK-F: 5′-AAAACCCACGTCCAACTCG-3′	PK-R: 5′-GGGTAAAGACCGTTTGCTGAT-3′
LDH-F: 5′-ATTTTAGTCGGTGATGGTGCTG-3′	LDH-R: 5′-AGAAGTCCAAGGAGTTGCGTC-3′

#### Impacts of Vitamins on Gene Expression

Transcriptomic analysis was employed to determine the gene expression during cultured in vitamin-deficient medium. Analysis of genetic differences was performed by comparing the CK group with the vitamin deficiency group. Total RNA was extracted using Trizol method. RNA purity was checked using the NanoPhotometer^®^ spectrophotometer (Implen, Inc., United States). RNA integrity was assessed using the RNA Nano 6000 Assay Kit of the Agilent Bioanalyzer 2100 system (Agilent Technologies, United States). After RNA samples were qualified, the RNA was reverse-transcript into cDNA and constructed library and conducted sequencing. The clustering of the index-coded samples was performed on a cBot Cluster Generation System using TruSeq PE Cluster Kit v3-cBot-HS (Illumina).

Differential expression analysis of two conditions/groups (two biological replicates per condition) was performed using the DESeq R package (1.18.0). DESeq provides statistical routines for determining differential expression in digital gene expression data using a model based on the negative binomial distribution. The resulting *p*-values were adjusted using the Benjamini and Hochberg’s approach for controlling the false discovery rate. Genes with an adjusted *p*-value < 0.05 found by DESeq were assigned as differentially expressed.

The clustering of the index-coded samples was performed on a cBot Cluster Generation System using TruSeq PE Cluster Kit v3-cBot-HS (Illumina) according to the manufacturer’s instructions. After cluster generation, the library preparations were sequenced on an Illumina HiSeq platform and paired-end reads were generated. Gene Ontology (GO) enrichment analysis of differentially expressed genes was implemented by the GOseq R package, in which gene length bias was corrected. GO terms with corrected *p*-value less than 0.05 were considered significantly enriched by differential expressed genes. KEGG^1^ is a database resource for understanding high-level functions and utilities of the biological system, such as the cell, the organism and the ecosystem, from molecular-level information, especially large-scale molecular datasets generated by genome sequencing and other high-throughput experimental technologies). KOBAS software was performed to test the statistical enrichment of differential expression genes in KEGG pathways.

### High Density Culture of *Lactobacillus* Strains in Skim Milk

In order to evaluate the influence of vitamins on the proliferation of *Lactobacillus* strains, skim milk medium, which widely used for culture of *Lactobacillus* strains, was used. The details were as follows:

#### Determination of Vitamins in Skim Milk Medium

The concentrations of Vc, nicotinic acid, Vb_1_, Vb_2_, and Vb_6_ were determined by high-performance liquid chromatography (HPLC) (Ren et al., 2014), and the determination of Vb_9_, Vb_5_, and biotin concentrations was carried out by microbiological assay (Shi et al., 2002; Wang et al., 2002; Gao and Xu, 2003).

#### Proliferation of *Lactobacillus* Strains in Skim Milk

The control group and experimental group cultures were grown in a 5-L fermenter (Biotech-5BG, Shanghai Baoxing Biological Equipment Co., Ltd., Shanghai, China) in 12% skim milk medium, which consisted of 120 g/L skim milk solution, sterilized at 110°C for 20 min. Vc, Vb_5_, and biotin were added to skim milk culture medium in the experimental group, but not the control group. The strains were inoculated in skim milk medium according to the optimum culture conditions. The culture was inoculated at 5%, the pH was controlled at 6.5, the stirring speed was 85 rpm, and the culture was carried out at 37°C for 14 h without ventilation. In the prior fermentation stage, the viable count was measured every 4 h, and then measured every 2 h after 8 h fermentation. *L. helveticus* CGMCC 1.1877 and *L. bulgaricus* LB4 were also measured.

### Statistics

All data were expressed as the mean ± standard deviation (SD). The *in vitro* experiments were carried out at least three times independently. Paired-samples *t*-test (among two groups) and one-way ANOVA with Bonferroni’s *post hoc* test (among multiple groups) were performed with the SPSS analysis software, and any differences were considered significant at *p* < 0.05.

## Results

### Vitamin Requirements of Strain CICC 22171

The number of viable cells of the strain CICC 22171 in vitamin-deficient medium were less than the number of viable cells in CK group (data not shown). The CK group used complete CDM. The vitamin-deficient medium refers to a medium in which a specific vitamin was absent from the CDM. Biotin, Vc, and Vb_5_ group were significantly different with the CK group (*p* < 0.01). Therefore, it was suggested that these three vitamins were necessary for the growth of *L. helveticus* CICC 22171.

### The Mechanism of Vitamins on the Proliferation of Strain CICC 22171

#### The Enzyme Activity and Gene Expression of Glucose Metabolism

**FIGURE 1:**
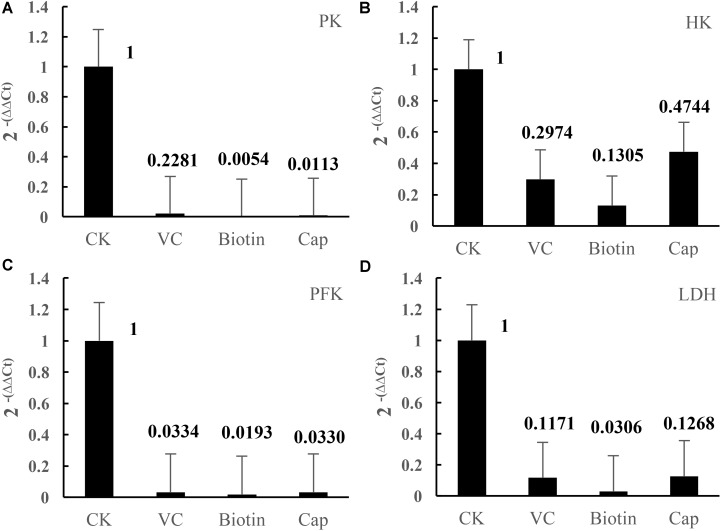
Effects of different vitamins on gene expression of key enzymes. **(A)** Pyruvate kinase–related gene expression in four groups. **(B)** Hexokinase-related gene expression in four groups. **(C)** Phosphofructokinase-related gene expression in four groups. **(D)** Lactate dehydrogenase–related gene expression in four groups. Data represent mean ± SD (*n* = 3 per group).

Compared with the CK group, the four enzymes, HK, PFK, PK, and LDH, were suppressed in vitamin-deficient medium (**Table 3**), especially in the Vc, biotin, and Vb_5_ vitamin-deficient medium groups, where the enzyme activities were significantly lower than those of the other groups, indicating that the three vitamins had a greater impact on the glucose metabolism and energy metabolism of strain CICC 22171. Additionally, four related genes were significantly downregulated in the Vc, biotin, and Vb_5_ deficient groups (**Figure 1**).

**Table 3:** Enzyme activity assay results.

Variable	Vb_1_	Vb_2_	Vb_6_	Vb_9_	PABA	NA	Vc	Biotin	Vb_5_	CK
HK (U/mg prot)	142.04	112.53	38.57	106.88	123.05	122.99	108.27	182.71	57.68	195.45
PFK (U/mg prot)	23.673	26.953	19.021	24.393	21.738	19.436	31.506	22.139	30.876	60.839
PK (U/mg prot)	17.819	32.553	34.827	36.423	63.763	39.653	19.428	23.004	20.980	93.394
LDH (U/mg prot)	2.778	2.909	2.347	1.330	1.787	4.725	2.280	2.763	3.425	6.219

*The strain was cultured in CDM in this group. Each vitamin group was cultured in the corresponding vitamin-deficient medium.*

#### Transcriptomic Analysis

A comparison between the control group and Vb_5_ group revealed that among the genes annotated for GO analysis, 201 were upregulated and 193 downregulated in Vb_5_ compared to the control. Comparison of the control group and Vc group showed that 18 genes were upregulated, and 15 genes were downregulated. In the comparison of the CK group and biotin group, there were 16 upregulated genes and 9 downregulated genes. The details were as follows:

##### Gene expression affected by Vb_5_

**FIGURE 2:**
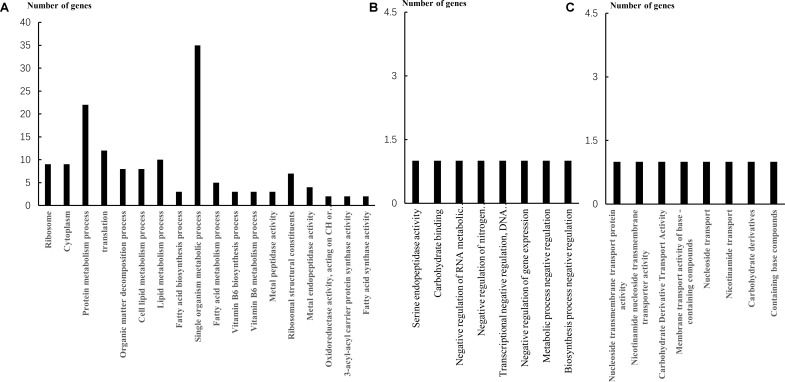
The down expressed genes in three group. **(A)** Downregulated genes of Vb_5_ in three categories. **(B)** Downregulated genes of Vc in three categories. **(C)** Downregulated genes of biotin in three categories.

According to the GO enrichment analysis, 32 genes were upregulated in the cellular component (CC) group, 19 genes were downregulated; 26 genes were upregulated in the molecular function (MF) group, 37 genes were downregulated; 143 genes were upregulated in the biological process (BP) group, 137 genes were downregulated. In the CC classification (**Figure 2A**), the intracellular ribosomes and cytoplasmic component-related genes of the cells were downregulated. In the BP classification, downregulated genes are mainly concentrated in the metabolism of proteins, fatty acids, intracellular compounds, and vitamins, including fatty acid and cell lipid metabolism process. In the MF classification, several enzyme activity–related genes and transcriptomic-related genes were downregulated. On the other hand, the upregulated genes were mainly focused on the transport of substances. KEGG analysis (**Table 4**) revealed that the downregulation pathway was associated with biotin metabolism, fatty acid synthesis, and metabolism, but cells increased the transport of nutrients such as sugar, protein, amino acids, and other nutrients, by upregulating the pathway of ABC transporter.

**Table 4:** Significant enrichment pathways.

	Downregulated	Upregulated
Vb_5_	Fatty acid metabolism	ABC transporters
	Fatty acid biosynthesis	
	Biotin metabolism	
Vc	Biotin metabolism	Purine metabolism
	Fatty acid biosynthesis	Biosynthesis of secondary metabolites
	Fatty acid metabolism	
Biotin	Biotin metabolism	Purine metabolism
		Biosynthesis of secondary metabolites

##### Gene expression affected by Vc

The GO enrichment analysis revealed that the downregulated genes were mainly related to carbohydrate binding and serine endopeptidase activity in the MF category (**Figure 2B**). In the BP annotation, the transcription and gene expression of negative regulatory–related genes was mainly downregulated. The upregulated genes were mainly associated with purine synthesis. The downregulation of the KEGG pathway was essentially the same as the Vb_5_ group, but the difference was that the Vc group ensured the activity of the strain by increasing the transcription and replication of the cells.

##### Gene expression affected by biotin

Downregulated genes were predominantly classified in the categories of nucleoside transmembrane transporters, nicotinamide nucleoside transmembrane transporters, and carbohydrate derivative transport activity in the MF annotations, which could affect the intracellular transport mechanism of bacteria (**Figure 2C**). Also, in the BP annotations, the downregulated genes were mainly related to nucleoside transport, carbohydrate derivative transport, and nitrogen transport, similar to the MF classifications (**Figure 2C**). Differentially expressed genes in this group also involved in fatty acid synthesis (**Table 5**) and the upregulated genes were associated with purine synthesis. The KEGG pathway analysis of the biotin group was the same as that of the VC group, as discussed above.

**Table 5:** Differentially expressed genes.

	Downregulated genes	Upregulated genes
Vb_5_	3-Hydroxyacyl-ACP dehydratase	
	3-Hydroxyacyl-ACP dehydratase	
	3-Ketoacyl-ACP reductase	Esterase
	3-Oxoacyl-ACP synthase	Esterase
	3-Oxoacyl-ACP synthase III	ATP-binding protein
	Biotin biosynthesis protein BioC	ABC transporter
	Acetyl-CoA carboxylase biotin carboxylase subunit	ABC transporter ATPase and permease
	Acetyl-CoA carboxylase biotin carboxyl carrier protein subunit	ABC transporter ATPase and permease
	Acetyl-CoA carboxylase carboxyltransferase subunit beta	ABC transporter ATP-binding protein
	Acetyl-CoA carboxyltransferase subunit alpha	ABC transporter permease
	GTPase Der	ABC transporter permease
	30S ribosomal protein S12	Methionine ABC transporter substrate-binding protein
	30S ribosomal protein S16	Sugar ABC transporter substrate-binding protein Sugar transporter
	30S ribosomal protein S7	
	30S ribosomal protein S9	
Vc	3-Hydroxyacyl-ACP dehydratase	Phosphoribosylformylglycinamidine synthase subunit PurL
	3-Ketoacyl-ACP reductase	Phosphoribosylaminoimidazole carboxylase
Biotin	3-Hydroxyacyl-ACP dehydratase	Phosphoribosylformylglycinamidine synthase subunit PurL
		Phosphoribosylaminoimidazole carboxylase

### High Density Culture of *Lactobacillus* Strains in Skim Milk

#### Determination of Vitamins in Skim Milk Medium

In the previous process, the quantity of the three vitamins added were Vc, 2 mg/L; Vb_5_, 1 mg/L; and biotin 1 mg/L. The content of these three vitamins in skim milk was determined (**Table 6**). The concentration of Vc in skim milk was very low; the content of Vb_5_ in skim milk was higher than in the defined medium; and the content of biotin was 0.0455 mg/L in skim milk, significantly less than the concentration added to defined media. Thus, biotin was added as a major vitamin to the skim milk medium.

**Table 6:** Vitamin contents in skimmed milk culture medium.

Vitamins	Reporting limit	Result
Vb_1_ (mg/100 g)	0.1	Absent
Vb_2_ (mg/100 g)	0.1	0.245
Vb_5_ (mg/100 g)	0.04	12.3
Vb_6_ (μg/100 g)	30	Absent
Vb_9_ (μg/100 g)	0.16	0.67
Vc (mg/100 g)	2	Absent
Nicotinic acid (μg/100 g)	70	Absent
Biotin (μg/100 g)	0.08	4.55

#### Proliferation of *Lactobacillus* Strains in Skim Milk

**FIGURE 3:**
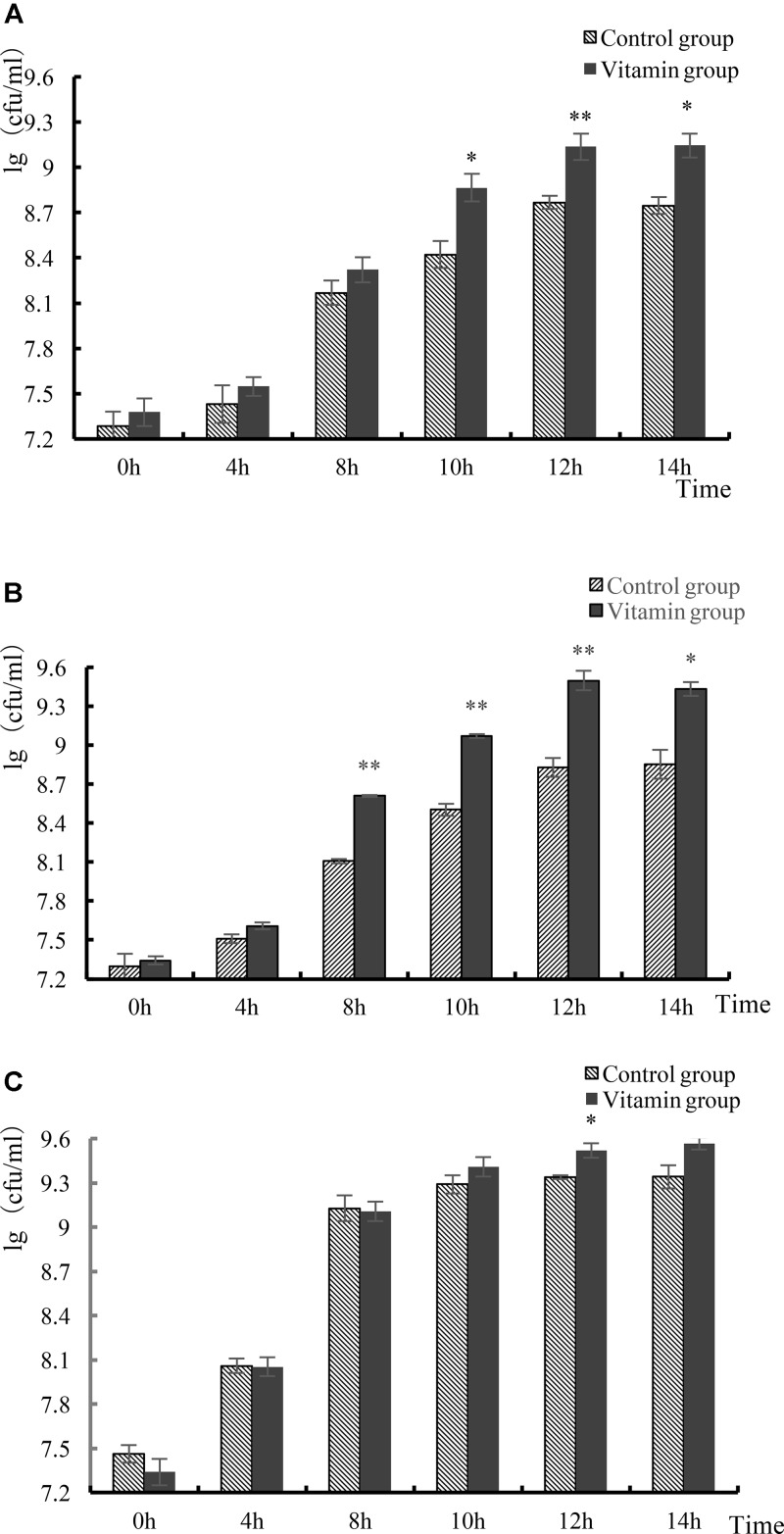
Growth of three strains. **(A)** Growth of *L. helveticus* CICC 22171 strain. **(B)** Growth of *L. helveticus* CGMCC 1.1877 strain. **(C)** Growth of *L. helveticus* LB4 strain. Data represent mean ± SD (*n* = 3 per group). Asterisks indicate values that are statistically different from control (^∗^*p* < 0.05, ^∗∗^*p* < 0.01).

The number of living cells of *L. helveticus* cultured in skim milk was generally no more than 10^9^ cfu/mL. After 12 h, the growth of the strain reached stationary phase, the phase that has the largest number of viable bacteria (**Figure 3**). The final viable count of *L. helveticus* CICC 22171 was 1.4 × 10^9^ cfu/mL in the experimental group and 5.87 × 10^8^ cfu/mL in the control group. The number of viable bacteria of *L. helveticus* CGMCC 1.1877 was 3.18 × 10^9^ cfu/mL in the experimental group, compared with 6.87 × 10^8^ cfu/mL in control group. Moreover, the viable count of *L. bulgaricus* LB4 was 3.34 × 10^9^ cfu/mL, but 2.18 × 10^9^ cfu/mL in the control group. By analysis and comparison, there was no significant difference between the two groups in the *L. bulgaricus* group (*p* > 0.05). The number of viable cells of the three strains were all higher than that of the control group. This demonstrated that vitamins could promote the growth of *L. helveticus* CICC 22171 and *L. helveticus* CGMCC 1.1877. However, these three vitamins had no significant effects on the proliferation of *L. bulgaricus.*

## Discussion

The important role of vitamins in the growth of LAB has been reflected in previous studies. Some *Lactobacillus plantarum* strains were found to be auxotrophic with respect to the Vb_6_, p-aminobenzoic acid, and nicotinic acid (Ruiz-Barba and Jiménez-Díaz, 1994). This study confirmed that vitamins play an important role in the growth of *L. helveticus.*

The HK, PFK, PK, and LDH play important roles in glycolytic pathway of the cell. The enzymatic activity and expression of four enzymes can reflect the growth status of the cells. The results indicated that the three vitamins had a greater impact on glucose metabolism and energy metabolism of strain CICC 22171. To determine the effect of different vitamins on the transcription of glucose-related genes in *L. helveticus*, the expression of four enzyme activity–related genes was measured. Gene expression results were consistent with the previous results of enzyme activity.

The Vb_5_ was discovered by Williams in 1933; it is a water-soluble vitamin and an essential nutrient for microorganisms. Vb_5_ is involved in the synthesis of coenzyme A (CoA) (Peterson and Peterson, 1945). The acyl group carrier of acetyl-CoA and acyl carrier protein (ACP) is CoA. Vb_5_ is also a coenzyme in a variety of enzyme reactions, mainly involved in protein metabolism and lipid metabolism *in vivo* (Huang and Wang, 1998). In various downregulated genes, ribosomes form the core of the translation machinery. Cytoplasm is the main site of metabolism; a great number of chemical reactions are carried out in the cytoplasm, such as glycolysis, amino acids, fatty acids, and nucleotide metabolism. Therefore, the function of ribosomes and cytoplasm is vital to cell growth (Wittmann, 1971; Lafontaine and Tollervey, 2001). Fatty acids play a key role in energy transport and storage, cell structure, intermediates that provide hormone synthesis, and so on. The main component of the cell membrane is fatty acids (Zhang and Rock, 2008). The decrease of cell lipid metabolism and protein metabolism was reflected in the previous enzyme activity assay (**Table 3**). The activities of oxidoreductase, metallopeptidase, transferase, fatty acid synthase, and ACP synthase were inhibited. Oxidoreductase can catalyze the oxidation or reduction of the substrate, and the electron donor or acceptor is required during the reaction. Oxidoreductase participates in a wide variety of catalytic reactions in the organism. Metal peptidase is a class of protease, which catalyzes the hydrolysis of peptide bonds in peptides and proteins. This indicates that the protein metabolism process has been inhibited and is consistent with the results of BP classification. Regarding upregulated genes, ABC transporters are members of a transport system superfamily (Tillotson and Tillotson, 2010). ATP binding and hydrolysis provide energy to support ABC transporters to transport various substrates across cellular membranes. In prokaryotes, importers transport the nutrients into the cell. Ions, amino acids, peptides, sugars, and other molecules that are mostly hydrophilic can be transported (Davidson et al., 2008). Two upregulated genes are related to sugar transport: as the fatty acid synthesis–related gene of *L. helveticus* is downregulated, the transport of nutrients was increased to ensure the growth of cells. In addition, upregulation of the esterase gene increases the efficiency of the use of fatty acids. In Vb5-deficient medium, the most important downregulated genes are genes associated with fatty acid synthesis. Fatty acid synthase is a multi-functional enzyme system, including ACP, ACP dehydratase, ACP reductase, ACP synthase, and some other enzymes (Chan and Vogel, 2010). Downregulation of the genes associated with the fatty acid synthase demonstrates that the growth of *L. helveticus* in the culture medium is inhibited (**Table 5**). Acetyl-CoA carboxylase (ACC) is a biotin-dependent enzyme. Providing the malonyl-CoA substrate for the biosynthesis of fatty acids is the most important function of ACC. One of the downregulated genes is associated with biotin synthesis, and the corresponding ACC-related genes are also downregulated. In the protein synthesis, the relevant genes have also been inhibited. Guanosine triphosphatases (GTPases) have been demonstrated to play a role in proper assembly of ribosomes in bacteria (Karbstein, 2007). Ribosomal protein genes are also inhibited, so the translation process of *L. helveticus* is blocked and therefore protein synthesis is adversely affected. It can be confirmed that fatty acid synthesis and protein synthesis of *L. helveticus* have been greatly inhibited in the absence of Vb_5_.

In the Vc group, there were serine endopeptidase-related genes in the downregulated group of genes. Serine protease is a class of important proteolytic enzymes that break the peptide bonds in macromolecules and generate small molecule proteins. It plays an important and extensive physiological role in the biological organism. Similar to the Vb_5_ group, the downregulated genes were associated with fatty acid synthesis (**Table 5**). Purines (adenine and guanine), as a constituent of DNA, ensure the normal DNA replication of the cells. Apart from this, purines are also significant components in many other important biomolecules, such as ATP, cyclic AMP, NADH, and CoA. Free radicals can destroy the body’s DNA, tissues, and mitochondria, and have negative impacts on the gene expression process. Vc is an antioxidant that protects organisms from free radicals. Therefore, purine synthesis–related genes may be associated with the antioxidant properties of Vc.

Biotin is a water-soluble vitamin belonging to the B-complex group. Biotin may be associated with the synthesis of various transporters in the cell. It can be therefore considered that the absence of biotin may lead to the inefficiency of material transport in the cell. The role of biotin in fat synthesis has been reported in the previous Vb_5_ group. Interestingly, the uptake gene is also related to the synthesis of purines. Biotin is involved in Vc biosynthesis and is therefore also associated with antioxidant activity. Studies have shown that biotin is associated with DNA replication. Therefore, it is not difficult to explain why upregulated genes are associated with purine synthesis (Walash et al., 2008).

To verify whether the proliferation of vitamins have species and genus differences, two strains of *L. helveticus* and one strain of *L. bulgaricus* which widely used in yogurt fermentation were used in skim milk fermentation. Vitamins not only promoted the proliferation of *L. helveticus* CICC 22171, but also promoted the proliferation of *L. helveticus* CGMCC 1.1877, indicating that Vb_5_, Vc, and biotin could promote the growth of *L. helveticus.* However, the growth promotion of *L. helveticus* was limited.

Vitamins are micronutrients essential for the metabolism of all living organisms. They are found as precursors of intracellular coenzymes that are necessary to regulate vital biochemical reactions in the cell. However, some LAB are auxotrophic so must ingest vitamins from the outside to support growth and metabolism. Some studies have shown similar results to this study, finding that vitamins can promote LAB proliferation. Biotin, 4-aminobenzoic acid, and thiamine accelerated the reproduction of *L. delbrueckii* and improved the yield of lactic acid (Li et al., 2001). The lactic acid production by *Lactobacillus paracasei* (*L. paracasei*) increased by 42.77% compared with the control group after the addition of vitamins, and glycolytic pathway and homologous lactic acid fermentation pathways were strengthened promoting the primary metabolism of *L. paracasei* (Qiu et al., 2007). It was found in another study that vitamins added to soytone could improve the efficiency of lactic acid production from glucose and lactose (Kwon et al., 2000).

## Conclusion

Vb_5_, Vc, and biotin were found to be necessary for the growth of *L. helveticus* and affected the expression of the main enzymes of glucose metabolism. Genetic differences analysis showed that these three vitamins were related to the synthesis of fatty acids in *L. helveticus* CICC 22171 and participate in the energy supply of the cells. Moreover, a cell proliferation test in skim milk culture medium confirmed that the addition of these three vitamins can promote *L. helveticus* proliferation, but this cannot be extended to *L. bulgaricus.* In summary, it was suggested that Vb_5_, Vc, and biotin were essential for the growth of *L. helveticus.* Having proven the important role of vitamins, it is possible to provide high-density cultivation of *L. helveticus* in skim milk, and provide theoretical guidance for the production of fermentative agents for *L. helveticus*.

## Author Contributions

JC, TW, and CY performed the laboratory work. CY wrote the manuscript and performed the data analysis. HZ provided some ideas and checked the manuscript. BZ provided some suggestions.

## Conflict of Interest Statement

The authors declare that the research was conducted in the absence of any commercial or financial relationships that could be construed as a potential conflict of interest.

## Footnotes

1 http://www.genome.jp/kegg/
